# Small Cell Carcinoma of the Gallbladder: Case Report and Comprehensive Analysis of Published Cases

**DOI:** 10.1155/2015/304909

**Published:** 2015-12-28

**Authors:** Carolyn Carrera, Paul Kunk, Osama Rahma

**Affiliations:** Division of Hematology and Oncology, Department of Medicine, University of Virginia Health System, Charlottesville, VA 22908, USA

## Abstract

*Background*. Gallbladder small cell carcinoma is a rare and highly aggressive malignancy with no established standard of care treatment. We described here a case report of small cell gallbladder cancer and we then performed a comprehensive review of 72 case reports of this disease.* Methods*. Published case reports of small cell carcinoma of the gallbladder between 1983 and 2014 were reviewed. Treatment modalities and survival were analyzed for metastatic and localized disease.* Results*. Median overall survival for all patients was 13 months. Metastatic disease was identified in 72% of cases. Treatment of metastatic disease with chemotherapy showed a significant survival benefit (*p* < 0.001) compared to no chemotherapy, and the use of platinum doublet with etoposide showed a nonsignificant 4-month improvement in survival compared to other chemotherapy regimens (*p* = 0.13). Adjuvant therapy did not demonstrate an improvement of median overall survival in local disease (*p* = 0.78).* Conclusion*. Given the limited available data, systemic therapy with platinum and etoposide should be considered for patients with metastatic small cell carcinoma of the gallbladder. Adjuvant chemoradiation or chemotherapy for treatment of local disease warrants further investigation.

## 1. Introduction

Small cell carcinoma of the gallbladder is a rare and highly aggressive malignancy that was first described by Albores-Saavedra in 1981 [1, 2]. While small cell carcinoma of the lung comprises approximately 15% of all lung cancers [3, 4], extrapulmonary small cell carcinoma is rare with a reported incidence of 0.1–0.4% [5, 6]. Similar to other extrahepatic biliary tumors, small cell cancer of the gallbladder is associated with cholelithiasis [7] and rarely with paraneoplastic syndromes including Cushing syndrome and paraneoplastic sensory neuropathy [8, 9]. Early metastases to regional and distant lymph nodes, liver, and lungs are common with 66% of patients having evidence of metastatic disease at diagnosis [7]. Median overall survival is reported to be approximately 9 months [6, 7].

Gross pathologic features of gallbladder small cell carcinoma include large tumor size with extensive necrosis and submucosal invasion [10]. Histological features show a proliferation of small round or oval cells arranged in sheets with scant cytoplasm and hyperchromatic nuclei with cytoplasmic neurosecretory granules under electron microscopy [11–13]. Small cell tumors typically express neuron-specific enolase (NSE), while some express synaptophysin, chromogranin, cytokeratin, and Leu-7 [13–15]. Similar to gallbladder adenocarcinoma, there is a high frequency of p53 and p16INK4a mutations [15].

No standard of care exists for treating gallbladder small cell carcinoma. While the overall survival benefit of chemoradiation or chemotherapy has been extrapolated from a meta-analysis of patients with high-risk biliary tract cancers [16], such benefit in patients with small cell histology remains unclear. Published treatment regimens for gallbladder small cell carcinoma are highly variable, including resection, radiation, and/or a variety of chemotherapy regimens [7].

Here, we present a case of metastatic gallbladder small cell carcinoma. We subsequently performed a comprehensive analysis of published case reports of gallbladder small cell carcinoma with an emphasis on the varying treatment strategies.

**Figure 1:**
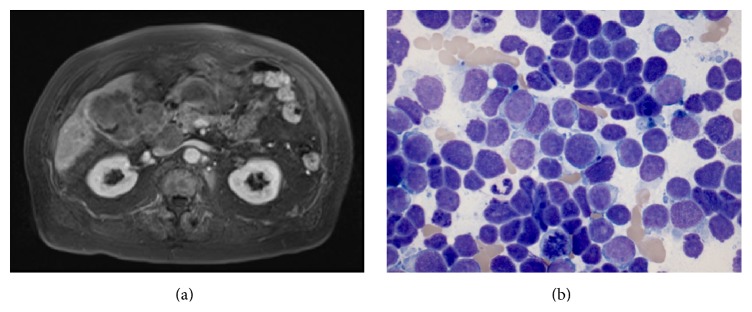
Patient case MRI and FNA of liver lesion. (a) MRI of the abdomen showed a bulky gallbladder tumor measuring 5.6 cm with direct extension into the liver, innumerable hepatic and periportal nodal metastases which exerted mass effect on the cystic duct, and peripancreatic and right diaphragmatic nodal involvement. (b) Ultrasound-guided biopsy of a metastatic liver lesion shows small and fragile epithelioid cells with little cytoplasm and fine chromatin. Abundant apoptotic bodies and mitotic figures were seen consistent with small cell carcinoma.

An 89-year-old man with a history of dementia and multiple myeloma presented with one month of right-sided abdominal pain. Noncontrasted CT scan of the abdomen revealed a 3 × 4 cm gallbladder mass with probable hepatic extension and enlarged portacaval, cystic, and peripancreatic nodes. MRI of the abdomen confirmed a bulky gallbladder tumor measuring 5.6 cm with direct extension into the liver. Innumerable hepatic and periportal nodal metastases with mass effect on the cystic duct, as well as peripancreatic and right diaphragmatic nodal involvement, were also seen (Figure 1). CT scan of the chest showed several indeterminate pulmonary nodules, mediastinal adenopathy, and spinal lytic lesions, but no primary lung mass. An ultrasound-guided FNA of one of the liver lesions showed small and fragile epithelioid cells with little cytoplasm and fine chromatin in addition to abundant apoptotic bodies and mitotic figures (Figure 1). Given the presence of gallbladder mass and subsequent FNA results, the diagnosis of gallbladder small cell carcinoma was made. Due to the patient's poor functional status and multiple medical comorbid conditions, he elected to pursue hospice care and passed away 3 months after diagnosis.

## 2. Materials and Methods

Case reports between 1983 and 2014 were obtained by searching PubMed using terms of “small cell carcinoma of the gallbladder” and “oat cell carcinoma of the gallbladder.” Patients were classified as having local (involving immediate regional lymph nodes or direct extension into the liver) or metastatic disease (involving distant lymph nodes, satellite liver metastasis, or other organ metastases). We favored staging this disease in a similar manner to small cell lung cancer rather than traditional TNM staging for cholangiocarcinoma given their similar histopathology and aggressive clinical course. Median overall survival was determined for the local and metastatic groups, as well as those with local disease who underwent adjuvant chemotherapy and/or radiation. Patients with metastatic disease were further classified with respect to the chemotherapy regimens they received and analyzed for survival benefit. Autopsy cases and clinical cases that did not report survival data were reported here but excluded from statistical analysis. Kaplan-Meier curves were developed, and *p* < 0.05 was considered statistically significant.

## 3. Results

### 3.1. Patient Characteristics

Seventy-two patients with small cell carcinoma of the gallbladder were identified [1–38]. Characteristics of clinical cases are listed in Tables 1 and 2, and autopsy cases are depicted in Table 3. Overall, this included 49 female and 23 male patients with an average age of 62.8 years (range 25–89). Nineteen of these patients were identified at autopsy, leaving 53 patients available for analysis.

### 3.2. Local Disease

Of the 53 patients analyzed, 20 patients (38%) were identified as having local disease (19 clinical cases and 1 autopsy case). The autopsy case was excluded from data analysis due to lack of survival data (Table 3). As shown in Table 1, average age of patients with local disease was 63 years (range 25–86), and local disease was more common in women (F : M of 13 : 6). Presenting symptoms in this group included abdominal pain, nausea, vomiting, jaundice, pruritus, and weight loss. The most frequently encountered histopathology was small cell carcinoma, but mixed histopathology of small cell carcinoma with adenocarcinoma, adenosquamous, undifferentiated carcinoma with endocrine features, and clear cell carcinoma was identified. Cholelithiasis was identified in 9/16 (56%) patients with local disease.

Treatment of local disease was variable. Eighteen of the nineteen analyzed patients (95%) underwent surgical resection, while only one patient did not undergo cholecystectomy due to extensive fibrosis and adhesions found intraoperatively [23]. Adjuvant treatment with chemotherapy and/or radiation was identified in 10/18 (56%) patients. Most frequently administered chemotherapy regimens included cisplatin/etoposide (4) and 5-FU (2). One patient received etoposide alone [14]. One patient received cyclophosphamide, vincristine, procarbazine, and prednisolone [22]. Two patients underwent chemotherapy with unnamed agents [24, 25]. Five patients underwent radiation to the liver or gallbladder fossa, with one receiving it for bone metastases in the setting of disease recurrence at 24 months [31].

### 3.3. Metastatic Disease

Metastatic disease accounted for 52 (72%) of the cases. Twenty-two of these 52 patients (42%) were found at autopsy or had unavailable survival data, and therefore they were excluded (Table 3). Thus, only 34 cases with metastatic disease were available for analysis (Table 2). Average age of patients with metastatic disease was 61.5 years (range 32–89), and metastatic disease was more common in women (F : M of 23 : 11). Presenting symptoms in this group were similar to those with local disease and included abdominal pain, nausea, cholestatic jaundice, and weight loss. The most frequently encountered histopathology was small cell carcinoma, but mixed histopathology with adenocarcinoma and intestinal metaplastic epithelium was also seen. Cholelithiasis was identified in 9/17 (53%) patients with metastatic disease. The most common sites of metastases included liver and distant lymph nodes (e.g., retroperitoneal, peripancreatic, hilar, and para-aortic). Other reported metastatic sites included the abdominal wall, omentum, pancreas, adrenal glands, small bowel, and colon.

Administered chemotherapeutic regimens in patients with metastatic disease consisted of platinum agent in combination with etoposide in the majority of the cases (7) followed by 5-FU (2) and gemcitabine/cisplatin (2). One patient received single agent carboplatin [7]. Two patients received adriamycin, vincristine, cyclophosphamide, and nitrosourea [2]. Two patients were identified as having undergone radiation therapy for retroperitoneal disease [31]. One patient was excluded from further analysis due to receiving multiple treatment regimens including platinum/etoposide and 5-FU administered closely together [32].

### 3.4. Autopsy Cases

Nineteen autopsy cases were identified (Table 3). Eighteen patients (95%) were found to have distant metastases to lymph nodes, liver, lung, peritoneum, pancreas, ovary, adrenal glands, and colon. Autopsy cases were predominately found to be earlier studies with the majority being published before 1990. These cases were excluded from further analysis due to lack of prognostic data.

### 3.5. Outcomes

**Figure 2:**
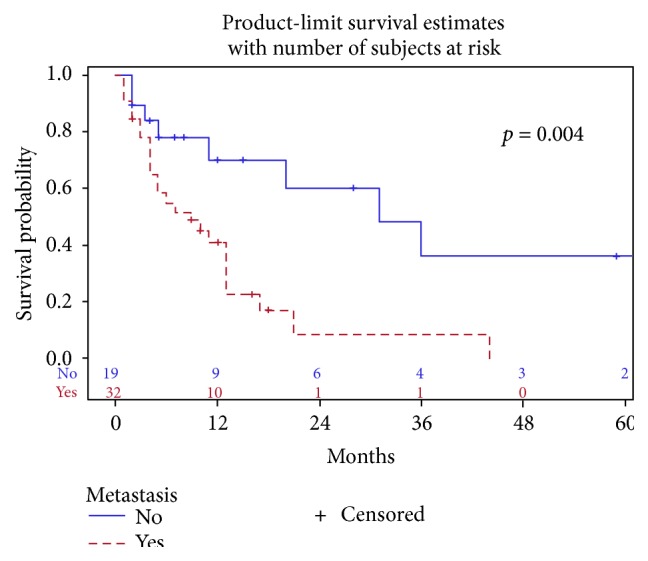
Survival for metastatic versus local disease.

Median survival for all patients was 13 months. Patients with local disease demonstrated a statistically significant improvement in median overall survival compared to those with metastatic disease (31 versus 9 months, resp., *p* = 0.004) (Figure 2). We were unable to report progression free survival, response, and recurrence rates due to unavailable data from the case reports.

**Figure 3:**
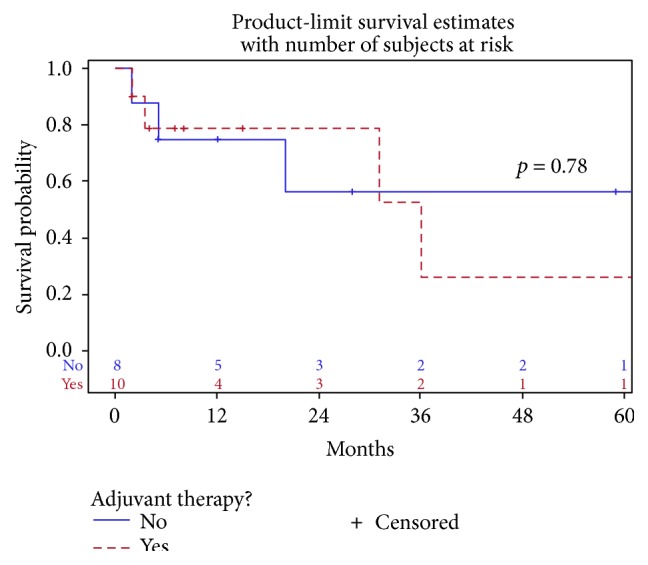
Survival for local disease adjuvant therapy versus no adjuvant therapy.

The vast majority of patients with local disease were treated with surgical resection. However, one patient did not undergo cholecystectomy due to extensive fibrosis and adhesions. The patient subsequently received chemoradiation with cisplatin/etoposide and survived 11 months [23]. There were two patients reported to have long-term survival within the local disease group. The first patient survived at least 189 months after surgical resection and adjuvant chemotherapy with a 5-FU-based regimen. The patient had lung and intra-abdominal recurrence but was reported to be alive at the time of publication [31]. The other patient survived more than 144 months after surgical resection without adjuvant treatment [21]. Based on our analysis adjuvant therapy was not found to improve median overall survival (*p* = 0.78) in patients with local disease (Figure 3).

**Figure 4:**
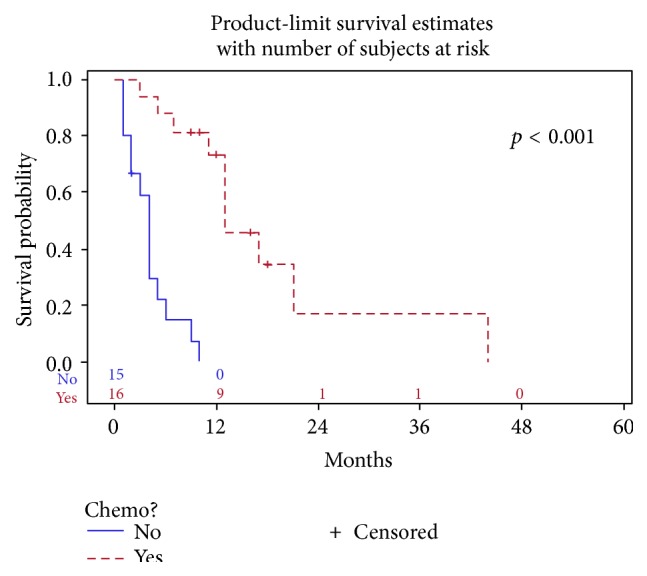
Use of chemotherapy for patients with metastatic disease.

**Figure 5:**
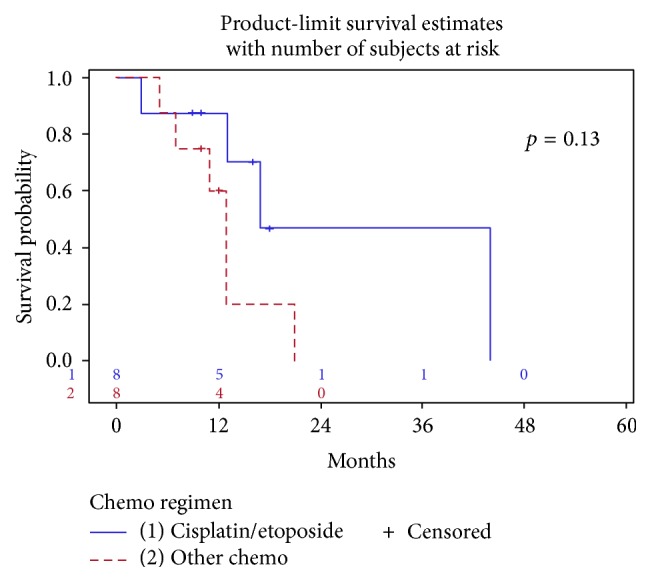
Chemotherapy regimen in patients with metastatic disease.

In contrast, patients in the metastatic group who received chemotherapy had statistically improved median overall survival compared to patients who had no chemotherapy (13 versus 4 months, *p* < 0.001) (Figure 4). In a subgroup analysis of patients with metastatic disease who received chemotherapy, the regimen of platinum/etoposide demonstrated a nonsignificant improvement in median overall survival compared to all other chemotherapy regimens (17 versus 13 months, *p* = 0.13) (Figure 5). Two patients with metastatic disease underwent radiation for retroperitoneal lymph node involvement with reported survival of 13 and 21 months [31]. Statistical analysis was not performed on this group of patients who received radiation therapy given its small sample size.

## 4. Discussion

We performed a comprehensive analysis of 72 cases of gallbladder small cell carcinoma. We found this malignancy to be reported most often in older patients and women. The majority of patients (72%) were found to have metastatic disease at the time of diagnosis, presumably due to the nonspecific presenting symptoms. We found the median overall survival to be 13 months, which is consistent with previously published reports (range of 9–31 months) [3, 6, 7, 31]. Not surprisingly, patients with metastatic disease had worse prognosis compared to those with local disease and benefited from treatment with chemotherapy, mainly platinum and etoposide combination. While our analysis showed no benefit of adjuvant chemotherapy in local disease, a trend toward improved survival was previously established with a multidisciplinary approach involving surgery, adjuvant platinum-based chemotherapy, and radiation therapy (21 months) compared to surgery alone (4 months), adjuvant chemotherapy alone (12 months), and chemotherapy alone (9 months) [6].

The rarity of gallbladder small cell carcinoma and the retrospective nature of our study pose significant limitations to the interpretation of our report. The disease has only been identified relatively recently [1] with only a few published case reports. Furthermore, data on response rates and progression free survival was unavailable from the published case reports, limiting analysis to overall survival benefit.

Small cell cancer of the lung has been studied more extensively given its higher incidence compared to small cell cancer of the gastrointestinal (GI) tract. Treatment of small cell carcinoma of the lung currently consists of chemoradiation in combination with platinum agents and etoposide for limited stage disease and chemotherapy for extensive stage disease based on large clinical studies [39–42]. Given the similar pathology and perhaps biology of small cell cancer of the lung and GI tract, it is not surprising that platinum agents in combination with etoposide provided the most survival benefit (although not statistically significant) in this report.

Due to the low incidence of gallbladder small cell carcinoma, it is unlikely that randomized clinical trials can be feasible to establish the standard treatment of this disease. Accordingly, future clinical trials could be designed to include patients with small cell histology regardless of the tumor origin. Future directions should also focus on better understanding of the molecular biology of these tumors in order to develop more effective therapies. Novel targets such as Bcl-2, VEGF, and delta-like protein 3 (DLL3) are currently being investigated in small cell lung cancer, which, if successful, may be applied to non-small-cell lung cancers [43, 44]. In summary, small cell cancer of the gallbladder represents a clinical challenge in daily practice given the paucity of available clinical data and the lack of randomized studies supporting the analysis of case reports as a clinically meaningful tool.

## Figures and Tables

**Table 1:** Clinical characteristics in local disease.

	Year	Age	Sex	Histology	Stones	Surgery	Adjuvant chemotherapy	Adjuvant radiation	Survival	Ref.
1	1988	72	F	SCC	No	Yes	None	No	2	[17]
2	1988	68	F	SCC	Yes	Yes	None	No	>59	[17]
3	1990	72	F	SCC	Yes	Yes	None	No	>12	[18]
4	1993	74	M	SCC	N/A	Yes	None	No	>28	[19]
5	1993	61	M	SCC	Yes	Yes	Etop.	No	2	[14]
6	1994	71	F	SCC, adenosquamous	Yes	Yes	None	No	20	[20]
7	1998	76	F	SCC	No	Yes	None	No	>144	[21]
8	1999	57	F	SCC	No	Yes	5-FU	Yes	31	[31]
9	1999	40	M	SCC, moderately differentiated adenocarcinoma	Yes	Yes	5-FU	No	>189	[31]
10	1999	79	M	SCC	No	Yes	Cyclophos., Vinc., Procarb., Pred.	No	3.5	[22]
11	1999	86	F	SCC	Yes	Yes	None	No	5	[22]
12	2000	25	F	SCC	N/A	Yes	None	Yes	>7	[10]
13	2000	81	F	SCC	Yes	Yes	None	No	>5	[12]
14	2002	67	M	SCC, undifferentiated with neuroendocrine features	Yes	No	Cis., Etop.	Yes	11	[23]
15	2002	66	F	SCC, clear cell carcinoma, and adenocarcinoma	Yes	Yes	Chemo N/A	No	36	[24]
16	2009	48	F	SCC	No	Yes	Cis., Etop.	No	>4	[9]
17	2010	47	M	SCC	No	Yes	Cis., Etop.	Yes	>8	[25]
18	2010	41	F	SCC	No	Yes	Chemo N/A	No	>2	[25]
19	2010	70	F	SCC	N/A	Yes	Cis., Etop.	Yes	>15	[6]

SCC = small cell carcinoma; Etop. = etoposide; Cyclophos. = cyclophosphamide; Vinc. = vincristine; Procarb. = procarbazine; Pred. = prednisolone; Cis. = cisplatin; chemo N/A = chemotherapy (agents not identified).

**Table 2:** Clinical characteristics in metastatic disease.

	Year	Age	Sex	Histology	Mets. site	Stones	Chemotherapy	Radiation	Survival	Ref.
1	1981	58	F	SCC	Hilar LN	Y	None	N	4	[1]
2	1984	79	F	SCC	Regional LN, liver	Y	None	N	4	[2]
3	1984	55	F	SCC	LN, liver	Y	Adr., Vinc., Cyclophos., Nitro.	N	11	[2]
4	1984	N/A	F	SCC	Regional LN, liver	Y	None	N	4	[2]
5	1984	52	F	SCC	Liver	N	Adr., Vinc., Cyclophos., Nitro.	N	13	[2]
6	1988	44	M	SCC	LN, liver, omentum, and colon	N	None	N	>2	[17]
7	1988	75	F	SCC	Liver	N	None	N	9	[17]
8	1988	56	F	SCC	Liver, abdominal wall	N	None	N	2	[17]
9	1991	71	F	SCC, adenocarcinoma	Liver, hilar LN, and peritoneum	N	None	N	4	[26]
10	1992	62	F	SCC	Liver, duodenum	N	None	N	5	[27]
11	1992	60	M	SCC	LN, liver	Y	Cis., Etop.	N	>18	[28]
12	1993	68	F	SCC	LN	N/A	None	N	2	[19]
13	1993	64	F	SCC	LN	N/A	None	N	6	[19]
14	1993	43	F	SCC	LN, liver	N/A	None	N	10	[19]
15	1993	57	M	SCC	Liver	N/A	None	N	N/A	[19]
16	1996	37	F	SCC	LN, liver, and omentum	N/A	Cis., Etop.	N	13	[29]
17	1996	82	M	SCC	LN, liver	N/A	None	N	1	[29]
18	1998	72	M	SCC	Liver	N/A	Platinum, Etop.	N	>16	[11]
19	1998	76	F	SCC, intestinal metaplastic epithelium	LN, liver	N/A	None	N/A	N/A	[30]
20	1999	69	F	SCC, poorly diff. adenocarcinoma	LN, liver, small bowel, and omentum	N	Cis., Etop.	N	44	[31]
21	1999	69	M	SCC	RP LN	Y	Strep., 5-FU	Y	21	[31]
22	1999	71	F	SCC, poorly diff. adenocarcinoma	RP LN, liver, and peritoneum	Y	5-FU	Y	13	[31]
23	2001	62	M	SCC	LN, liver	Y	Cis., Etop.	N	17	[3]
24	2001	56	M	SCC	Liver	Y	5-FU, Cis., Doce., Carbo.	N	13	[32]
25	2001	70	F	SCC	Liver	N/A	None	N	1	[33]
26	2005	48	M	SCC	LN, liver	N/A	Gem., Cis.	N	7	[34]
27	2005	51	M	SCC	LN, liver	N/A	Gem., Cis.	N	5	[34]
28	2005	47	F	SCC	Liver	N/A	Pac, Ifos., Cis.	N	>12	[35]
29	2005	49	F	SCC	Liver	N/A	Pac, Ifos., Cis.	N	>10	[35]
30	2006	77	F	SCC	LN, liver	N/A	Chemo N/A	N	N/A	[36]
31	2008	76	F	SCC	LN, liver	N/A	Cis., Etop.	N	>10	[37]
32	2011	54	F	SCC	LN, liver	N	Carbo.	N	3	[7]
33	2009	32	F	SCC	LN, liver	N/A	Cis., Etop.	N	>9	[15]
34	2015	89	M	SCC	LN, liver, and lung	N	None	N	3	*∗*

SCC = small cell carcinoma; LN = distant lymph nodes; RP = retroperitoneal; Adr. = adriamycin; Vinc. = vincristine; Cyclophos. = cyclophosphamide; Nitro. = nitrosourea; Cis. = cisplatin; Etop. = etoposide; Dox. = doxorubicin; Mito. = mitomycin; Doce. = Docetaxel; Carbo. = carboplatin; chemo N/A = chemotherapy (agents not identified); Pac = paclitaxel.

*∗* = present case.

**Table 3:** Autopsy cases.

	Year	Age	Sex	Histology	Metastasis	Mets. site	Stones	Ref.
1	1981	55	M	SCC	Y	LN, liver	Y	[1]
2	1981	72	F	SCC	Y	LN, liver	Y	[1]
3	1981	74	F	SCC	Y	LN, lung	Y	[1]
4	1981	65	M	SCC	Y	LN, liver, peritoneum, and pleura	Y	[1]
5	1984	75	F	SCC	Y	LN, liver, and lung	Y	[2]
6	1984	67	F	SCC	Y	LN, liver, and lung	Y	[2]
7	1984	66	F	SCC	N	LN	Y	[2]
8	1984	67	F	SCC	Y	LN, liver, peritoneum, and splenic vein	Y	[2]
9	1984	60	F	SCC	Y	LN, liver, and lung	Y	[2]
10	1984	60	F	SCC	Y	LN, liver, ovary, and peritoneum	Y	[2]
11	1984	48	F	SCC	Y	LN, liver, and lung	Y	[2]
12	1984	72	F	SCC	Y	LN, liver	Y	[2]
13	1984	55	F	SCC	Y	LN, liver, and omentum	Y	[2]
14	1984	50	F	SCC	Y	LN, liver	Y	[2]
15	1988	50	F	SCC	Y	LN, biliary duct, liver, pancreas, duodenum, and colon	N/A	[17]
16	1988	69	M	SCC	Y	LN, liver, biliary duct, duodenum, and lung	Y	[17]
17	1988	73	M	SCC	Y	LN, liver, and pancreas	Y	[17]
18	1991	70	M	SCC, adenocarcinoma	Y	LN, liver, rectum, pancreas, and adrenal	N/A	[38]
19	1993	83	M	SCC	Y	LN, liver, and pancreas	N/A	[19]
